# Heat-Transfer Properties of Additively Manufactured Aluminum Lattice Structures in Combination with Phase Change Material

**DOI:** 10.3390/ma17071672

**Published:** 2024-04-05

**Authors:** Immanuel Voigt, Rico Schmerler, Hannes Korn, Welf-Guntram Drossel

**Affiliations:** 1 Professorship Adaptronics and Lightweight Design, TU Chemnitz, Reichenhainer Straße 70, 09126 Chemnitz, Germany; welf-guntram.drossel@mb.tu-chemnitz.de; 2 Fraunhofer Institute for Machine Tools and Forming Technology IWU, Nöthnitzer Straße 44, 01187 Dresden, Germany; rico.schmerler@iwu.fraunhofer.de (R.S.); hannes.korn@iwu.fraunhofer.de (H.K.)

**Keywords:** laser powder bed fusion, micro-lattice, heat transfer, experiment, FEM

## Abstract

Compared to sensible heat storage, latent heat storage provides higher energy density due to the enthalpy difference of the storage medium undergoing a phase change. However, the heat storage capability of phase change materials is opposed by low thermal conductivity. To enable sufficient heat transfer within a latent heat storage unit, phase change materials can be used in combination with a metallic matrix. One approach is the infiltration of phase change materials into additively manufactured metallic lattice structures. In this work, the fabrication of aluminum lattice structures through laser powder bed fusion is described. During fabrication, the cell size and the strut diameter were varied to obtain specimens of different geometries. To obtain the thermal conductivity of the fabricated lattices, measurements were conducted based on the transient plane source method. Additionally, finite element simulations were carried out to evaluate the effect of fabrication and measurement uncertainties. The thermal conductivity of the fabricated lattices was found to be between 3 W/(m·K) and 130 W/(m·K). The numerically and analytically performed calculations provide good estimations of the experimentally obtained data.

## 1. Introduction

Phase change materials (PCMs) offer an effective way of storing and releasing heat as the temperature during phase transition remains almost constant. PCMs make use of the phase transition enthalpy occurring upon melting and solidification. As an energy-efficient thermal management tool, PCMs are used in a wide range of applications such as temperature control in buildings and photovoltaic systems as well as for thermo-regulation in textiles [1,2,3,4]. However, the direct use of PCMs for latent heat storage systems is often hindered due to their low thermal conductivity. The thermal conductivity of organic PCMs is found to be in the range between 0.1 W/(m·K) and 0.4 W/(m·K), while inorganic PCMs exhibit values between 0.5 W/(m·K) and 1.3 W/(m·K) [1]. Numerous studies address this topic by proposing the combination of PCMs with highly conductive materials. To enhance the effective thermal conductivity of a latent heat storage system, carbon nanofiber, carbon nanotubes, and silver nanoparticles can be used [5,6]. Another approach is the infiltration of PCMs into open-cell or closed-cell aluminum foams [7,8]. While the composite structures of metallic foam and PCMs that are investigated in the literature demonstrate convenient thermal performance, there are drawbacks due to the limitations of the foam fabrication. For example, the foam porosity as a main composite parameter cannot be freely adjusted. Another drawback is the random distribution of pores within the foam structure.

As an alternative to foam structures, additively manufactured micro-lattice structures can be used. The main advantage of additively manufactured micro-lattice structures is the high fabrication flexibility and local adaptivity of the lattice density. With respect to micro-lattice–PCM composites, the increased geometric flexibility leads to an easier optimization process of the relation between matrix density and heat storage volume. Takarazawa et al. investigated the heat-transfer and pressure-drop characteristics of micro-lattices [9]. Different lattice block designs were compared. It could be shown that the face-centered cubic (FCC) lattices exhibit superior effective thermal conductivity compared to the body-centered cubic (BCC) lattices. Bracconi et al. analyzed the effective thermal conductivity of isotropic and anisotropic lattice structures by means of finite element (FE) models [10]. The authors stated that the effective thermal conductivity is not affected by varying unit cell size and hence mainly depends on the porosity and the thermal conductivity of the solid. Experimental investigations on heat sinks fabricated using laser powder bed fusion (LPBF) were presented by Wong et al. [11]. Different heat sink designs, including one lattice structure, were fabricated and experimentally compared regarding their heat-transfer and pressure-drop characteristics. The convective heat dissipation on the lattice structure was found to be inferior compared to the other heat sinks.

While the stated studies demonstrate several influences of geometric parameters on the heat-transfer characteristics of additively manufactured lattice structures, additional examinations are required to fully comprehend the effects of LPBF on the effective thermal conductivity of the lattices for different strut diameters and unit cell sizes. In particular, the thermal performance of lattice structures filled with PCMs has not yet been addressed in the literature and thus needs to be examined. Therefore, this paper deals with the LPBF fabrication and thermal characterization of regular lattice structures of different geometric parameters. The transient plane source (TPS) method was used to obtain the effective thermal conductivity of the manufactured specimens. The measurements were conducted for air-filled and PCM-filled lattices. The measurements were accompanied by FE simulations to interpret the experimental results and detect possible uncertainty effects. The investigations addressed the following aspects:

- Influence of LPBF process parameters on the resulting lattice geometry;
- Comparative TPS measurements of thermal conductivity of air-filled and PCM-filled lattice structures;
- Estimation of heat-transfer characteristics of manufactured lattice structures using analytical and numerical calculations.

## 2. Materials and Methods

In the following paragraphs, the design and fabrication of selected lattice structures are described. The experimental set-up and test procedure for obtaining the thermal conductivity are illustrated. Furthermore, the thermal FE model of the lattice structures is presented.

### 2.1. Lattice Structure Design and Manufacturing

**Figure 1:**
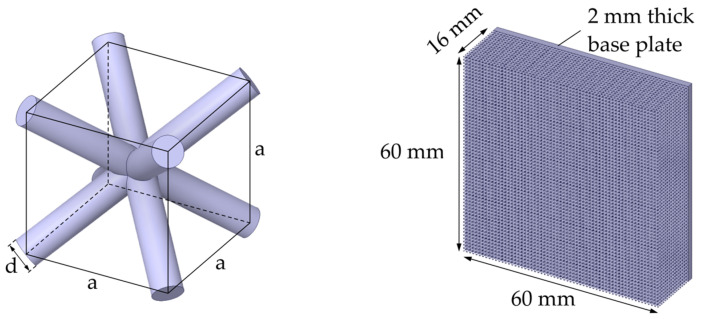
BCC unit cell and specimen dimensions. Geometric parameters strut diameter *d* and unit cell size *a* are labelled.

Regular lattice structures with BCC unit cells were chosen for the experimental and numerical investigations. By using BCC unit cells, good comparability with other studies on additively manufactured metallic lattice structures is achieved, as BCC unit cells are often considered [12,13,14,15]. In a BCC lattice structure, all struts exhibit the same inclination angle to the base plate during manufacturing (approx. 35°), which leads to a homogeneous strut thickness [13]. Furthermore, the relative density can be changed over a particularly wide range using this unit cell type [16,17]. Based on the BCC unit cell, lattice structures with a bounding box of 60 mm × 60 mm × 16 mm were considered. In addition to the lattice structure, the specimen geometry consisted of a base plate with a thickness of 2 mm and a cross section of 60 mm × 60 mm. The unit cell and the complete specimen geometry are shown in Figure 1.

The relative density $\rho_{rel}$ of a BCC lattice is defined by the ratio of the strut diameter to the cell size d/a and can be calculated using the polynomial approximation given by Zhang et al. [18]

(1) $$\rho_{rel,est}=\frac{V_{lat}}{V_{tot}}=-0.0014+0.01097\left(\frac{d}{a}\right)+5.4142{\left(\frac{d}{a}\right)}^{2}-4.8785{\left(\frac{d}{a}\right)}^{3},$$

where $V_{lat}$ is the lattice volume and $V_{tot}$ the enclosing cuboid volume.

The specimens were additively manufactured through LPBF using the alloy AlSi10Mg, according to ASTM F3318 [19], by the manufacturer Concept Laser (Lichtenfels, Germany), with the following particle size distribution: Q3, 10% = 19.3 µm; Q3, 50% = 28.8 µm; and Q3, 90% = 46.9 µm (measured using the particle analyzer Camsizer X2, Retsch Technology, Haan, Germany). The as-built specification of heat conductivity was 125 W/(m·K). This value was determined as the average of three measurements using the TPS method. The LPBF machine used was a Lasertec12 by DMG Mori/Realizer (Bielefeld, Germany) from 2020. The machine specifications are shown in Table 1.

The purpose of the investigations was to find dependencies between the effective thermal conductivity and the cell size a of the unit cells as well as the strut diameter d. Therefore, the size of the unit cells and the laser parametrization during manufacturing were varied using a D-optimal experimental design, resulting in different strut diameters. The parameters were chosen in such a manner to cover a wide range of $\rho_{rel}$. Table 2 lists the fabricated specimens and the corresponding geometry parameters. The corresponding laser parameters used for obtaining the different lattice structures are shown in Table 3. The selection of the laser parameters was based on the results of comprehensive preliminary manufacturing experiments that are not within the focus of the present study.

Two specimens of each geometric configuration were considered as required by the experimental method for obtaining the effective thermal conductivity. The strut diameter for each specimen was estimated by adjusting the corresponding parameter within the CAD model until the measured effective density coincided with the virtual effective density. This estimation assumed an ideal lattice topology with constant strut diameter. The obtained strut diameter values were validated through a microscopic examination of the specimens. Furthermore, the estimation of the relative density by means of Equation (1) was found to be in good agreement with the relative density $\rho_{rel,meas}$ obtained by weighing the specimens.

**Figure 2:**
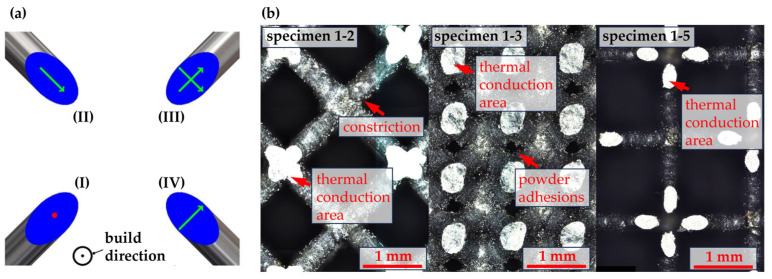
(**a**) Different possible scanning strategies considered during manufacturing: (**I**) point-like scanning, (**II**) line scanning, (**III**) cross scanning, and (**IV**) transversal scanning. (**b**) Detailed view of the struts of the manufactured specimens.

As a special scanning strategy, the quasi-point scanning strategy was used for the manufacturing of the lattice section of the specimens [20]. Instead of scanning a contour along the cross section of the struts and filling it using hatches, the cross sections were exposed using two crossed scanning vectors (cross, see Figure 2a). This very defined energy input leads to very thin and even struts in the lattice structure [21]. Figure 2 depicts the different scanning strategies as well as a detailed view on the resulting struts in the manufactured specimens. The even areas on the outer specimen surfaces can easily be seen due to the high reflectivity (see Figure 2b). The lattice geometry is varied in a wide range depending on the geometric parametrization. The resulting struts are even and free of major defects. However, depending on the manufacturing parametrization, the struts can exhibit minor defects, like constrictions at the junction between struts and nodes or powder adhesions on the struts.

### 2.2. Infiltration with Phase Change Material

**Figure 3:**
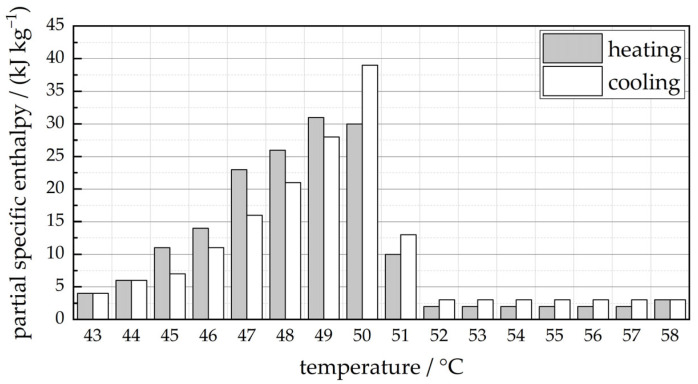
Partial specific enthalpy of PCM RT50 according to manufacturer data [22].

Typical PCMs can be categorized into organic and inorganic PCMs. Since inorganic PCMs such as salt hydrates and fatty acids are mostly not compatible with metals and exhibit non-congruent melting, an organic, paraffin-based PCM was used for the investigations. The PCM RT50 of the manufacturer Rubitherm Technologies (Berlin, Germany) was selected. RT50 exhibits a solid–liquid phase change temperature range between approximately 45 °C and 51 °C. The thermal conductivity of RT50 amounts to 0.2 W/(m·K), as stated by the manufacturer [22]. The temperature-dependent partial specific enthalpy values within the solid–liquid phase change are shown in Figure 3. The specific enthalpy difference for the temperature range of 43 °C to 58 °C, as stated by the manufacturer, amounts to 160 J/g. In the work of other authors such as Losada-Pérez et al., that comprises calorimetric measurements on similar paraffin-based PCMs of the same manufacturer, comparable, but slightly lower values are reported (e.g., 138 J/g for RT42 in the range of 34 °C to 51 °C) [23].

**Figure 4:**
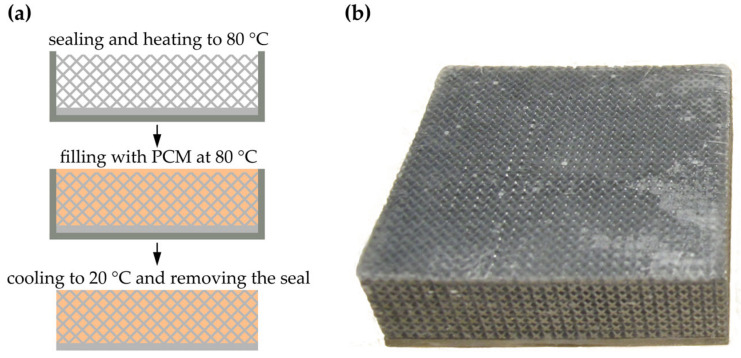
PCM infiltration method (**a**) and infiltrated specimen 2–7 (**b**).

The infiltration process is illustrated in Figure 4a. At first, the outer surfaces of the specimens were sealed using polyvinyl chloride (PVC) tape. To avoid a low infiltration ratio due to the premature solidification of the PCM upon contacting the lattice structure, the specimens were heated up to 80 °C using a convection oven. The PCM was molten at the same oven temperature. After a cooling period of over 15 h at an ambient temperature of 20 °C, the PVC tape was removed and the outer surfaces of the specimens were manually ground to remove PCM residuals outside of the defined specimen volume. An exemplary infiltrated specimen is shown in Figure 4b.

The heat-storage characteristics of the specimens can be described by means of the enthalpy difference ΔH with respect to a defined temperature range. To illustrate the influence of the relative lattice density on the storable heat within the air-filled lattices ($\Delta H_{al}$) and the PCM-filled lattices (${\Delta H}_{al-PCM}$), the enthalpy difference is calculated for all specimens based on the specific heat capacity of aluminum ($c_{p,al}=0.88$ J/(kg K)) and the specific enthalpy curve $\Delta h_{PCM}(T)$ of the PCM RT50 (see Figure 3) using

(2) $${\Delta H}_{al-PCM}={\Delta H}_{al}+{\Delta H}_{PCM}=c_{p,al}\;m_{al}\Delta T+\Delta h_{PCM}\;m_{PCM}.$$

The values are calculated considering an exemplary temperature rise from 43 °C to 58 °C, as listed in Table 4. Within the considered temperature range, the infiltrated specimens 1–5 and 2–5 exhibit the highest heat storability due to the low lattice density and thus high PCM mass.

### 2.3. Heat-Transfer Investigation through TPS Method

The TPS method is described in DIN EN ISO 22007-2 [24]. The purpose of the TPS method is the temperature-dependent determination of the thermal conductivity for various materials, ranging from insulating materials with very low thermal conductivity to materials with very high conductivity, such as copper. The further description of the TPS method is taken from the manual of the TPS instrument manufacturer Hot Disk^®^ (Göteborg, Sweden) [25]. The measuring range of the TPS method is given as [0.005, 1800] W/(m·K). The TPS method was selected as it meets the requirements for measuring porous metallic structures such as good thermal contact and proper measurement area to cover a representative geometry or rather several pores. It has been proven as suitable in several investigations [26,27,28].

In the TPS method, a sensor made of electrically conductive nickel wire, which is in the form of a double spiral and embedded in polyimide foil, is positioned between two identical samples. The 60–80 µm thick sensor acts as a heat source to raise the sample temperature and, at the same time, as a resistance thermometer.

For a successful determination of the thermal characteristic values, restrictions for input variables, like measuring time, heat power, and relaxation time, and characteristic result variables, like temperature gradients and the penetration depth of the heat wave, must be observed. First, the specimens were measured without surface treatment. Afterwards, the specimens were ground to generate plane-parallel, flat surfaces and measured again. The grinding was performed using sandpaper of grit size P220. By comparing the results of the measurements before and after the grinding process, conclusions were aimed to be drawn regarding the required surface treatment of the specimens. Before the measurements, the specimens were conditioned for at least 24 h at room temperature.

**Figure 5:**
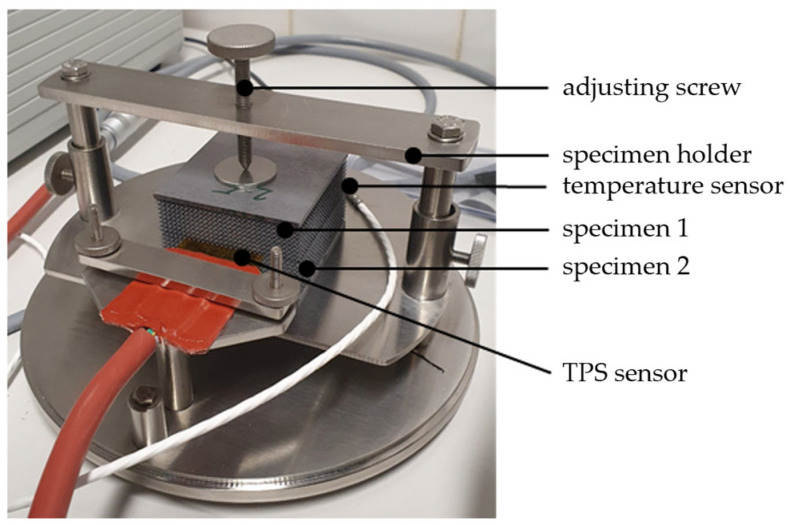
TPS measurement setup for thermal conductivity investigations.

The tests on the aluminum specimens were carried out with the aid of a HotDisk TP2500S measuring device (Hot Disk AB, Göteborg, Sweden). The experimental setup is illustrated in Figure 5. The sensor was positioned between two identical specimens. Slight pressure was applied to the samples and the sensor via a set screw to ensure good contact and thus heat conduction. The thin and flexible sensor allowed good thermal contact between the sensor and the sample. The use of a protective hood, not shown, reduced convective influences. The relaxation time in between measurements was at least 36 times the measurement duration.

The aluminum specimens were measured using the 3D bulk method. The cubic specimens were loaded with 0.15–4.5 W heating power within the measurement duration of 1–20 s; see Table 5. The radius of the sensor used was 9.87 mm.

To determine the PCM influence on the thermal conductivity of aluminum specimens, the seven specimen pairs were measured before and after infiltration with the PCM. Each measurement was made using two identical specimens, one placed above and one below the sensor, and taking the average. The measurement for each specimen pair was performed at least three times after the appropriate relaxation time. The infiltrated PCM was expected to have a neglectable influence on the heat-transfer characteristics of the composite structure due to the low thermal conductivity of the PCM. While the thermal conductivity of PCM is higher compared to air, the lack of convective heat transfer inside infiltrated lattices might affect the measurements. Furthermore, it was to be investigated if the continuous surface of the aluminum–PCM composite provides measurements of enhanced accuracy compared to the sole lattice contact areas due the enhanced heat input into the specimens.

### 2.4. Finite Element Simulation

Steady-state FE simulations were conducted aiming to evaluate the experimental results with respect to possible measurement uncertainties. Two FE models were built to simulate the thermal behavior of the manufactured lattice structures. The first model represents the sole aluminum lattice structure. The second model comprises the lattice structure with infiltrated PCM. The differential equation to be solved is the steady-state Laplace equation without internal heat generation:

(3) $$k\left(\frac{\partial^{2}T}{\partial x^{2}}+\frac{\partial^{2}T}{\partial y^{2}}+\frac{\partial^{2}T}{\partial z^{2}}\right)=0,$$

where k is the thermal conductivity. The assumed thermal conductivity for aluminum and PCM amounts to $k_{al}=$ 125 W/(m·K) and $k_{PCM}=$ 0.2 W/(m·K), respectively. The boundary conditions are given by the heat input on the bottom side of the specimen

(4) $$-k{\left.\frac{\partial T}{\partial z}\right|}_{\Gamma_{A}}=q_{\mathrm{in}}$$

and a homogenous temperature on the upper side of the specimen

(5) $$T=20\;{}^{\circ}C\;on\;\Gamma_{B}.$$

**Figure 6:**
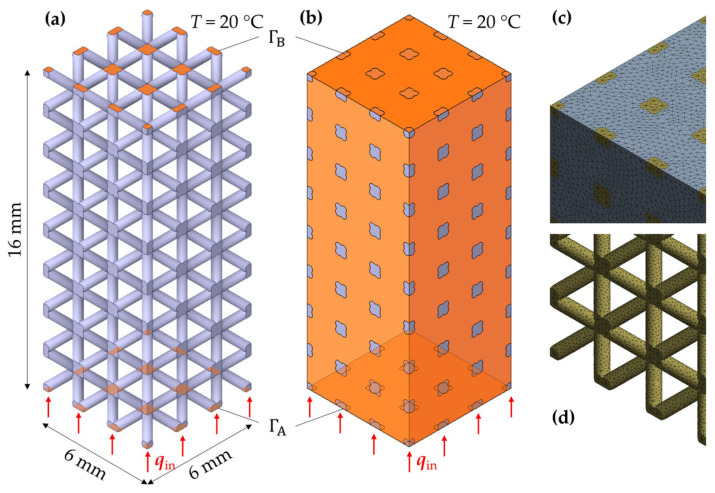
FE model of aluminum lattice (**a**) and lattice infiltrated with PCM (**b**), and corresponding meshes with PCM (**c**) and without PCM (**d**).

The commercial FE software Ansys (Release 2021 R2) was used to solve the thermal boundary value problem. The two FE models are illustrated in Figure 6 for exemplary parameter configurations. To reduce the model size, 10% of the specimen geometry is considered (6 mm × 6 mm × 16 mm). Ten-node tetrahedral elements (element type SOLID291) were used to discretize the geometry. For the aluminum–PCM composite, the contacts were defined considering perfect heat conduction at the contact surfaces (zero thermal contact resistance). A heat flow of 1 W was defined at $\Gamma_{A}$, which gave a heat flux of around 27.78 kW/m^2^.

The numerical results are used to calculate the effective thermal conductivity

(6) $$k_{e}=\frac{h\;q_{in}}{T_{A}-T_{B}}.$$

By means of $k_{e}$, the heat transfer of each lattice configuration can be quantized in dependency of the relative density $\rho_{rel}$. For $\rho_{rel}=1$, $k_{e}$ equals $k_{al}$, representing the thermal conductivity of a homogenous aluminum body.

### 2.5. Analytical Estimation

In addition to FE simulations, analytical models can be used to estimate the effective thermal conductivity of porous or lattice structures. While several approaches have been proposed in the literature, two models were considered in the present work, aiming for a comparison to the experimentally and numerically obtained values. As shown by various authors (e.g., Takezawa et al. [29]), the Hashin–Shtrikman (HS) bounds provide a convenient estimation for the effective thermal conductivity of two-phase composites [30]. The HS bounds can be calculated from

(7) $$k_{HS-}=k_{2}-\frac{3\;\rho_{rel}\;k_{2}\left(k_{1}-k_{2}\right)}{3\;k_{2}+\left(1-\rho_{rel}\right)\left(k_{1}-k_{2}\right)}$$

for the lower HS bound $k_{HS-}$ and

(8) $$k_{HS+}=k_{1}-\frac{3\;{(1-\rho }_{rel})\;k_{1}\left(k_{1}-k_{2}\right)}{3\;k_{1}+\rho_{rel}\left(k_{1}-k_{2}\right)}$$

For the upper HS bound $k_{HS+}$, with $k_{1}>k_{2}$.

As an estimate for open-cell and closed-cell metal foams, Ashby et al. introduced two bounds for the effective thermal conductivity that can be obtained by the inequation

(9) $${\rho_{rel}^{\;}}^{1.8}<\frac{k_{e}}{k_{s}}<{\rho_{rel}^{\;}}^{1.65},$$

where $k_{s}$ is the thermal conductivity of the foam material [31]. Ohsenbrügge et al. stated that the lower Ashby bound was found to be in good agreement with the effective thermal conductivity of the closed-cell aluminum foam examined in their work [32].

Both approaches were applied to the specifications of the fabricated specimens and evaluated with respect to the estimation quality.

## 3. Results

**Figure 7:**
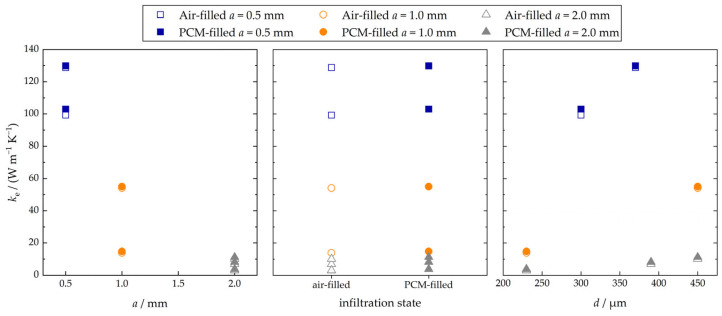
Experimentally obtained effective thermal conductivity values in dependency of the cell size, the infiltration state, and the strut diameter.

The effective thermal conductivity was obtained through measurements for the seven specimen pairs in air-filled and PCM-filled configurations. The measured values are illustrated in Figure 7 in dependency of the cell size, the infiltration state, and the strut diameter. It can be stated that, within the considered parameter limits, the effective thermal conductivity exhibits a high dependency on the cell size (negative correlation) and a moderate dependency on the strut diameter (positive correlation). Only slight differences in the effective thermal conductivity between air-filled and PCM-filled configurations were observed. The infiltration of the PCM led to an increase in the experimentally obtained effective thermal conductivity of a minimum of 0.8% for specimen pair 4 ($\rho_{rel}=0.99$) and a maximum of 25.3% for specimen pair 5 ($\rho_{rel}=0.07$). The lower the relative density of the lattice, the higher the observed relative increase in the effective thermal conductivity is.

The experimental results are listed alongside the numerically obtained results in Table 6. The comparison mainly demonstrates a good agreement between measurement and simulation data. For the specimen pairs 1, 3, 4, 5, and 6, the relative deviation is found to be below 10%. The relative deviation between measurement and simulation for specimen pairs 2 and 7 amounts to 28.8% and 23.1% for the air-filled configuration (17.5% and 17.8% for the PCM-filled configuration), respectively. Very high deviations of up to 53.8% occurred with respect to the measurements conducted prior to the grinding of the specimens (see $k_{\exp}^{pre}$ in Table 6). This implies poor initial contact conditions between the specimens and the TPS sensor due to the rough surface caused by the manufacturing process.

**Figure 8:**
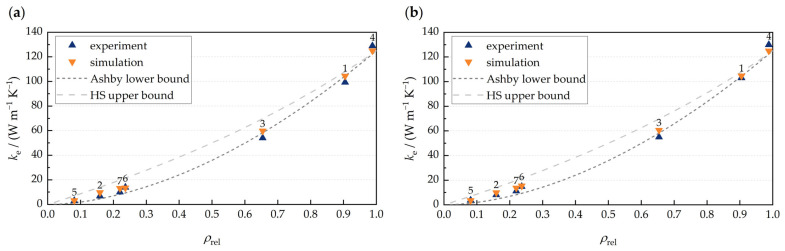
Experimentally and numerically obtained effective thermal conductivity in dependency of the relative density for air-filled (**a**) and PCM-filled lattices (**b**).

In Figure 8, the experimentally and numerically obtained values for the effective thermal conductivity are illustrated in dependency of the relative density. In addition, the Ashby lower bound and the HS upper bound are used to calculate the corresponding values. It can be stated that the Ashby lower bound provides a very good estimation for the effective thermal conductivity for $\rho_{rel}>0.6$. For $\rho_{rel}<0.3$, the experimentally and numerically obtained values are found between both bounds. The deviations in this range are over 28% for the HS upper bound and over 35% for the Ashby lower bound. Thus, a precise prediction of the effective thermal conductivity for the manufactured lattice structures cannot be achieved by means of the Ashby lower bound and HS upper bound.

Based on the measurements, an exponential fit was proposed to calculate the effective thermal conductivity using the function

(10) $$k_{e}\;/\;(Wm^{-1}K^{-1})=e^{A+B(d/a)+C{(d/a)}^{2}}$$

**Figure 9:**
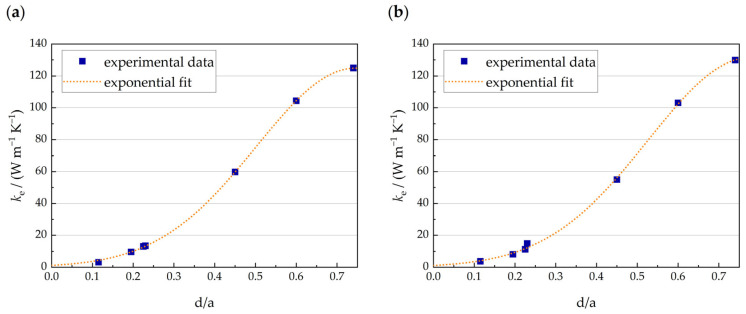
Exponential fits of the effective thermal conductivity obtained through measurements for air-filled (**a**) and PCM-filled lattices (**b**) as a function of the diameter-to-unit cell size ratio.

Using the Levenberg–Marquardt algorithm, the regression parameters were obtained with *A* = 0.12838, *B* = 12.607, and *C* = −8.4547 for the air-filled configuration and *A* = 0.092708, *B* = 12.297, and *C* = −7.8972 for the PCM-filled configuration. As illustrated in Figure 9, the regression functions represent the experimental data with high precision. The exponential fits exhibit coefficients of determination of $R^{2}=$ 0.99989 (air-filled) and $R^{2}=$ 0.99939 (PCM-filled).

## 4. Discussion

The experimental and numerical examinations of the heat-transfer characteristics of the fabricated lattices led to consistent findings on the effect of the varied geometric parameters. The highest deviations between the experimentally and numerically obtained results were found for specimen pairs 2 and 7, which exhibit a cell size of 2 mm and a low specific density of 0.16 and 0.22, respectively. Hence, the low contact surface areas between the specimens and the sensor are a potential cause of measurement inaccuracy. This assertion is affirmed by the increased agreement between simulation and experiment for specimen pairs 2 and 7 in the PCM-filled configuration, as the aluminum–PCM composite provides a continuous contact area and therefore potentially an enhanced heat transfer into the specimens. Another consequence of the infiltrated PCM is the prevention of convection effects on the inner surface of the lattice, potentially increasing the measurement precision.

The relevance of the quality of the outer specimen surfaces becomes apparent through the comparison of the experimental results obtained before and after the specimen grinding. This observation is due to rough specimen surfaces, leading to higher thermal contact resistance values and hence to an increased temperature drop at the specimen–sensor interface. Accordingly, the effective heat conductance values measured prior to grinding are significantly below the actual values. While the detailed examination of the surface roughness was not addressed in the present investigations, it can be stated that the specimen grinding with sandpaper of grit size P220 led to a significant increase in the measurement quality, resulting in a good agreement of experimental and numerical results.

The reproducibility of the measurements was ensured by measuring each specimen pair three times. The relative standard deviation was found to be below 1% for all specimen pairs, demonstrating a high degree of reproducibility.

The inaccurate prediction of the effective heat conductance by means of the FE model may be caused by irregularities of the fabricated lattices that are not considered within the CAD model. Such irregularities might occur due to constrictions and powder adhesions at the junctions. Lower or higher cross sections of the fabricated lattices may lead to underestimating or overestimating the effective heat conductance when considering a regular lattice geometry with a constant strut diameter. While the precise adjustment of the desired strut diameters during manufacturing still requires a preliminary process parameter identification, the reproducibility of the obtained geometries was found to be high, as observed using optical microscopy. This applies also to the occurrence of lattice irregularities such as powder adhesions with respect to different unit cells within one specimen as well as the comparison between different specimens. However, more detailed investigations, using methods like computed tomography scanning, need to be conducted to further evaluate the geometric accuracy of the manufactured specimens. It is expected that a comprehensive knowledge of the lattice diameter deviations leads to an increased prediction quality when using thermal FE models.

Furthermore, the results demonstrate that it is possible to predict the heat-transfer characteristics of lattice structures manufactured using LPBF by means of analytical approximations. For aluminum lattices with a specific density greater than 0.6, the Ashby lower bound was found to provide a convenient approximation. As the Ashby lower bound in the work of Ohsenbrügge et al. was considered a good estimation of the effective thermal conductivity of closed-cell aluminum foams [32], it can be stated that both structure types, aluminum BCC lattices and closed-cell aluminum foams, exhibit similar heat-transfer characteristics at a congruent specific density.

## 5. Conclusions

The present work deals with the thermal characterization of aluminum lattice structures. Regular aluminum lattice structures were manufactured using LPBF. The process parameters were varied to create lattice structures of different strut diameters and BCC unit cell sizes. The effective thermal conductivity of seven specimen pairs was obtained based on the TPS method. To assess the thermal characteristics of lattice structures as a composite matrix in latent heat storage systems, the specimens were infiltrated with a paraffin-based PCM. The measurements were repeated on the resulting composite structures. In addition to the measurements, the effective thermal conductivity of the lattices was obtained numerically by means of FE models.

The findings of the investigation can be summarized as follows:

- The experimental data demonstrate the influence of the strut diameter and the unit cell size on the heat-transfer characteristics of the manufactured lattices. The specimen infiltration with PCM led to a small increase in the effective heat conductivity in dependency of the lattice density. The experimentally obtained effective thermal conductivity can be formulated as an exponential function of the diameter-to-unit cell size ratio.
- During the measurements, the roughness of the outer specimen surface was found to have a high impact on measurement precision.
- The results obtained by means of FE simulation were mainly found to be in good agreement with the experimental data. For specimens with a low specific density, the simulations overestimated the measured effective thermal conductivity, implying a potential effect of lattice irregularities.
- The comparison of the measurement data with previously published findings indicates similar heat-transfer characteristics of aluminum BCC lattices and closed-cell aluminum foams.

With respect to the application of aluminum lattice structures in latent heat storage systems, a central design task is determining the appropriate lattice density that provides adequate heat transfer as well as sufficient volume for the PCM infiltration. As an increased density leads to an enhanced heat transfer and, at the same time, reduced latent heat storage volume, the optimum lattice characteristics depend on the corresponding case-specific requirements. The relationship between geometric and physical parameters obtained in the present study can be employed as a design tool for latent heat storage systems. Due to the geometrical freedom provided by the additive manufacturing of lattice structures, it appears possible to locally adjust the effective thermal conductivity, leading to spatially dependent and anisotropic heat transfer behavior. As a consequence, a more balanced compromise between heat transfer and heat storage performance can be obtained in comparison to conventional approaches that address the heat transfer enhancement in latent heat storage systems. The examination of corresponding lattice structures is suggested as a potential topic for future investigations.

## Figures and Tables

**Table 1:** Specification of the LPBF machine used for manufacturing the specimens.

	Parameter	Value
Laser	Optics	F-Theta
	Beam profile	Gaussian
	Max. power	400 W
	Min. focus	35 µm
Process	Atmosphere	Nitrogen
	Preheating	200 °C
	Recoating sequence	Bidirectional (along x-axis)
	Recoater type	Silicone lip
	Powder management	Closed loop
	Gas stream	Along y-axis
	Layer height	25 µm

**Table 2:** Fabricated specimens with corresponding geometric parameters.

Specimens	Parameter Set	a/mm	d/µm	$\rho_{rel,est}$	$\rho_{rel,meas}$
1-1 and 2-1	A	0.5	300	0.90	0.90
1-2 and 2-2	A	2.0	390	0.17	0.16
1-3 and 2-3	C	1.0	450	0.66	0.65
1-4 and 2-4	C	0.5	370	0.99	0.99
1-5 and 2-5	B	2.0	230	0.06	0.07
1-6 and 2-6	B	1.0	230	0.23	0.24
1-7 and 2-7	C	2.0	450	0.22	0.22

**Table 3:** Parameter sets with corresponding process parameters used during LPBF manufacturing.

Parameter Set	A	B	C
Laser power/W	200	150	180
Scan speed/(mm/s)	179	1000	257
Focus diameter/µm	150	55	55

**Table 4:** Enthalpy difference between 43 °C and 58 °C of the air-filled and PCM-filled lattices as well as corresponding lattice and PCM mass.

Specimens	$\rho_{rel,meas}$	$m_{al}$/g	$m_{PCM}$/g	$\Delta H_{al}$/J	${\Delta H}_{al-PCM}$/J
1-1 and 2-1	0.90	137	2	1808	2128
1-2 and 2-2	0.16	24	40	317	6717
1-3 and 2-3	0.65	98	15	1294	3694
1-4 and 2-4	0.99	147	0	1940	1940
1-5 and 2-5	0.07	11	47	145	7665
1-6 and 2-6	0.24	35	36	462	6222
1-7 and 2-7	0.22	33	37	436	6356

**Table 5:** TPS measurement parameters.

Specimens	Air-Filled, before Grinding		Air-Filled, after Grinding		PCM-Filled	
	Power/W	Time/s	Power/W	Time/s	Power/W	Time/s
1-1 and 2-1	0.90	3	2.20	1	3.00	1
1-2 and 2-2	0.15	5	0.30	4	0.60	10
1-3 and 2-3	1.00	3	1.80	2	3.00	2
1-4 and 2-4	1.60	1	3.30	1	4.50	1
1-5 and 2-5	0.15	5	0.30	4	0.50	20
1-6 and 2-6	0.30	3	0.50	2	0.50	2
1-7 and 2-7	0.15	5	0.50	3	0.50	3

**Table 6:** Experimentally and numerically obtained values for the effective thermal conductivity of the specimens in air-filled and PCM-filled configurations. $k_{\exp}^{pre}$ denotes the values measured prior to specimen grinding.

Specimens	Air-Filled			PCM-Filled	
	$k_{\exp}^{pre}$/(W/(m·K))	$k_{\exp}$/(W/(m·K))	$k_{\mathrm{sim}}$/(W/(m·K))	$k_{\exp}$/(W/(m·K))	$k_{\mathrm{sim}}$/(W/(m·K))
1-1 and 2-1	48.18	99.33	104.38	103.05	104.67
1-2 and 2-2	4.64	6.85	9.625	8.12	9.84
1-3 and 2-3	37.95	54.02	59.75	54.98	60.56
1-4 and 2-4	79.85	128.90	124.98	129.90	124.98
1-5 and 2-5	2.13	2.96	3.13	3.71	3.45
1-6 and 2-6	10.36	13.86	13.42	14.81	15.37
1-7 and 2-7	6.04	10.02	13.03	11.20	13.63

## Data Availability

The raw data supporting the conclusions of this article will be made available by the authors on request.
